# Chronic Macrophage Migration Inhibitory Factor Exposure Induces Mesenchymal Epithelial Transition and Promotes Gastric and Colon Cancers

**DOI:** 10.1371/journal.pone.0098656

**Published:** 2014-06-02

**Authors:** Katherine T. Morris, Robert A. Nofchissey, Irina V. Pinchuk, Ellen J. Beswick

**Affiliations:** 1 Department of Internal Medicine, Division of Gastroenterology and Hepatology, University of Texas Medical Branch, Galveston, Texas, United States of America; 2 Department of Surgery, University of New Mexico, Albuquerque, New Mexico, United States of America; 3 Department of Molecular Genetics and Microbiology, University of New Mexico, Albuquerque, New Mexico, United States of America; Rutgers - New Jersey Medical School, United States of America

## Abstract

Macrophage Migration Inhibitory Factor (MIF) is an inflammatory cytokine that is highly produced in gastrointestinal cancers. Since chronic inflammation is a risk factor for tumorigenesis in these cancers, in this study, the role of MIF in pro-tumorigenic events was examined. MIF and its receptor, CD74, were examined in gastric and colon tumors and found to be increased in most tumors with significantly higher expression in tumors from patients with lymph node metastasis. MIF was also found to be highly produced by cancer associated fibroblasts isolated from human tumors compared to fibroblasts from matched normal tissues from uninvolved areas. Fibroblast-produced MIF highly increased GI cancer cell proliferation, which was decreased upon neutralizing MIF or CD74. Chronic MIF treatment led to sustained proliferation and signaling events in non-transformed GI fibroblast cells, which was maintained upon removing MIF treatment for 8 weeks. Additionally, chronic treatment of normal GI cells expressing fibroblast markers for up to 16 weeks with MIF led to a drastic decrease of fibroblast markers with concurrent increase of epithelial markers. Transformation was examined by telomerase and focus forming assays. These results suggest the MIF promotes mesenchymal epithelial transition, cell transformation and tumorigenesis in GI cancers, and thus may be an important link between chronic inflammation and tumorigenesis.

## Introduction

Gastric and colorectal cancers are, respectively, the third and fourth leading causes of cancer related death worldwide [1]. Chronic inflammatory states such as *Helicobacter pylori* infection for gastric cancer and inflammatory bowel disease for colorectal cancer are key risk factors associated with the development of these malignancies [2]. Chronic inflammation is thought to have a role in carcinogenesis by increasing cell proliferation and cell resistance to apoptosis, and yet, a complete understanding of the underlying mechanisms by which inflammation and carcinogenesis are linked remains elusive.

MIF is an inflammatory cytokine that is highly produced in the gastrointestinal tract during inflammatory diseases. We have previously shown that it is highly produced by gastric epithelial cells when exposed to *H. pylori* and acts in an autocrine manner to induce transactivation of the epidermal growth factor receptor, which suggests an important link to pro-carcinogenic mechanisms [3], [4]. One study has also suggested a role for MIF in inflammatory bowel disease [5]. Other groups have shown MIF to be a potential diagnostic or prognostic marker in gastric and colon cancers [6]–[8]. Despite these associations, investigation of the mechanisms by which MIF is implicated in gastrointestinal cancers is not yet complete.

The primary receptor for MIF is CD74, or the major histocompatibility class II-associated invariant chain. The main role of CD74 was thought to be in the processing of class II MHC molecules on antigen presenting cells. However, the discovery of CD74 on the surface of epithelial cells suggests more complex activity of this receptor [9]. We have shown CD74 is important in proliferation of gastric carcinoma cells while others have suggested this in other inflammation-associated cancers such as prostate and renal cancers [10], [11]. Unregulated cell proliferation remains a hallmark of cancer, and given the known stimulation of several central pro-proliferation pathways MIF induces upon binding to CD74, further investigation into the role of MIF in inflammation-associated cancers is warranted.

In addition to the autocrine role of chronic inflammation in tumors, recent research into the involvement of stromal cells in the initiation and development of gastrointestinal cancers has revealed an important role for myofibroblasts (MF). MF are alpha-smooth actin^+^ (α-SMA), CD90^+^ stromal fibroblasts that have a critical role in initiation of tumors, as well as their growth and metastasis [12]. These cells are thought to facilitate tumor growth and invasion by producing pro-tumorigenic factors that act on tumor epithelial cells in a paracrine manner [12]. Given their location just underneath the epithelial layer in the gastrointestinal tract, and their role as key regulators of chronic inflammation, tumor growth, and metastasis, one goal of this study was to investigate the potential for MF to produce MIF in the tumor microenvironment.

Furthermore, recent discoveries of the ability of tumor cells to transition between an epithelial phenotype to a mesenchymal phenotype (EMT) in order to migrate and metastasize have also led investigators to consider that the reverse (mesenchymal to epithelial transition or MET) can occur when tumor cells extravasate into new sites and then need to set up a metastatic focus to survive [13]. This process involves a transition of spindle shaped mesenchymal cells to rounded epithelial cells that have tight junctions for cell to cell contact. Markers of the epithelial phenotype include epithelial antigen (EpCam) and the cell adhesion molecule, E-cadherin. Less is known about MET than is regarding EMT, but an emerging body of evidence suggests that MET may be a crucial step in both early tumor development as well as metastasis [13]. The signals which lead to the process of MET especially in gastrointestinal tract associated inflammation and cancer are unknown. In this study, we expanded on the known pro-tumorigenic properties of MIF to examine the role of MIF in MET in a chronic model.

The results of this study revealed that MIF and the MIF receptor (CD74) were highly expressed in human gastric and colorectal samples, with higher expression seen in more aggressive tumors from patients with nodal involvement. In addition, we found that tumor associated MF were a source of elevated MIF production when compared to MIF production from normal tissue associated MF and that conditioned media from tumor associated MF increased gastrointestinal carcinoma proliferation. This effect was greatly reduced with MIF blockade, suggesting that MIF is in large part responsible for the tumor progression due to MF cytokine production. Perhaps most intriguingly, however, was the demonstration that chronic MIF treatment led to changes in pro-tumorigenic signaling pathways in gastrointestinal carcinoma cells as well as MET in cells expressing fibroblast markers. The observed persisting chronic changes suggest a possible mechanism behind chronic inflammation and tumor progression, which may make MIF a therapeutic target for gastrointestinal cancers. This is the first study to show the direct impact of an inflammatory cytokine on MET and the transformation of normal cells.

## Methods

### Tissue Culture

HS738 non-transformed gastric/intestinal cells were purchased from American Type Tissue Culture (ATCC, Manassas, VA) and maintained in DMEM with 10% FBS, L-glutamine, and antibiotics. N87 gastric carcinoma cells and Caco-2 colon carcinoma cells were purchased from ATCC and maintained in RPMI with 10% FBS, 2 mM L-glutamine, and antibiotics, and MEM with 20% FBS, 2 mM L-glutamine, and antibiotics, respectively. Recombinant MIF was a kind gift from Dr. Richard Bucala, Department of Internal Medicine at Yale School of Medicine. Cells were treated with 10 ng/ml of MIF for up to 18 weeks. Morphology was photographed using a VWR Vista Vision microscope with digital camera attachment.

### Human Tissue Samples

Gastric and colon tissues were collected from normal (uninvolved) and tumor areas from discarded tissues from patients undergoing surgical resections. Epithelial cells from matched normal and cancer tissues were obtained by a series of 3 collagenase cell dissociations using the GentleMACS system (Miltenyi Biotech, Bergisch Gladbach, Germany). A cocktail of collagenase I, II, and IV were used in a 25000 U/ml stock solution of HBSS. Epithelial cells in the supernatants of digested tissues were incubated for 24 hours in non-adherent tissue culture plates to recover from enzymatic digestion. Epithelial cells were isolated from uninvolved normal tissues and tumor tissues as previously described and used for the flow cytometry analysis of CD74 expression [14]. Fibroblasts were isolated according to the protocol routinely used in our laboratory [15], [16]. The purity of isolated CD90^+^ (99%) was confirmed by flow cytometry. Cells were cultured in complete Modified Eagle Medium (MEM) with 10% FBS.

### Ethics Statement

Human tissue samples obtained at University of New Mexico Health Sciences Center were collected under human protocols approved by the UNMHSC Human Research Protections Office. Human tissue samples obtained at Legacy Research were collected under human protocols approved by the Legacy Research Institutional Review Board. Written consent was obtained using consent forms approved by each institutional review board.

### Real-Time PCR

RNA was isolated using trizol (Life Technologies, Grand Island, NY) according to the manufacturer’s instructions. RNA concentrations were measured using a Nanodrop instrument (Thermo Scientific, Wilmington, DE). Real-time PCR was performed according to Applied Biosystems’ two-step protocol (Applied Biosystems, Foster City, CA). All reagents were purchased from Applied Biosystems. The RT reaction mixture includes random 2.5 µM hexamers, 500 µM dNTPs, 0.4 U/µL of the RNase inhibitors, 5.5 mM MgCl_2_, MultiScribe Reverse Transcriptase (3.125 U/µL) and its buffer, and 1 µg of cellular RNA. The RT volume mix was adjusted to a final volume of 50 µL using RNase and DNase free H_2_O. The RT step was performed according to the following protocol: 10 min at 25°C, 60 min at 37°C, 5 min at 95°C. Obtained cDNA samples were stored at −80°C and used for the PCR reaction step. The PCR reaction mix was prepared using the Assays-on-Demand gene expression assay mix (Applied Biosystems) for human 18S, MIF, CD74, CD90, vimentin, EpCam, E-cadherin, and TERT (a 20X mix of unlabeled PCR primers and TaqMan MGB probe, FAM dye-labeled) and 2 µL of cDNA were added to the PCR reaction mix. The reaction was carried out according to the following protocol: 2 min at 50°C, 10 min at 95°C (1 cycle), and 15 sec at 95°C and one min at 60°C (45 cycles) on Applied Biosystems’ StepOnePlus instrument. The endpoint used in real-time PCR quantification, CT, was defined as the PCR cycle number that crossed the signal threshold. Quantification of cytokine gene expression was performed using the comparative CT method (Sequence Detector User Bulletin 2; Applied Biosystems) and reported as the fold difference relative to the human housekeeping gene, 18 S mRNA.

### Flow Cytometry

Immunostaining was performed according to standard FACS staining Biolegend protocols (Biolegend, San Diego, CA). For staining epithelial cells, the following antibodies were used; anti-EpCam Alexa-Fluor 488 clone 9c4 (Biolegend), anti-CD74 PE clone LN2 (Biolegend), and anti-E-cadherin efluor 660 (eBioscience, San Diego, CA). For fibroblast markers, the following antibodies were used; anti-CD90 PE clone 5E10 (Biolegend) and anti-vimentin FITC clone V9 (eBioscience). Manufacturer recommended isotype controls were utilized for each fluorochrome. All samples were run on a Guava easyCyte 8HT flow cytometer (Millipore, Bellerica, MA), and analyzed using FCS Express software (DeNovo Software, Los Angeles, CA).

### Luminex Bead Arrays

MIF was measured in supernatants by singleplex bead assay (Biorad, Hurcules, CA) according to manufacturer’s instructions. Signaling proteins, p-Akt, p-c-Jun, and p-Erk1/2, were measured by multiplex bead array by lysing 2×10^5^ cells with Bioplex lysis buffer kit and running assays according to manufacturer’s instructions on a Luminex 200 machine.

### Proliferation

HS738, N87 and Caco-2 cells (2500 per well) were added to wells of a 96 well plate and incubated with recombinant MIF or fibroblast supernatants for 48 hours. Proliferation was measured using CyQuant dye for DNA content (Life Technologies). Samples were read on a Tecan fluorescent plate reader (Mannedorf, Switzerland).

### Focus Forming Assays

HS738, N87, and Caco-2 cells were plated at 1×10^3^ in 100 mm plates and grown for 2 weeks with media changes 3 times a week. Cells were fixed in 10% formalin for 5 minutes. Formalin was decanted and 4 ml of Harris Modified hematoxylin was added to plates for 1 hr. Plates were incubated at room temperature in the dark. Three washes were performed with dH_2_0 and 1 ml of 10% NH_4_OH was added for 1 minute. After 2 more washes with dH_2_0, plates were air dried and colonies per plate counted.

### Statistical Analysis

Results were expressed as the mean ± SE of data obtained from at least three independent experiments done with triplicate sets in each experiment. Differences between means were evaluated by ANOVA using Student’s *t*-test for multiple comparisons. Values of *p*<0.05 were considered statistically significant.

## Results

### Human Gastric and Colon Tumors and Tumor Derived Fibroblasts Produce MIF

**Figure 1:**
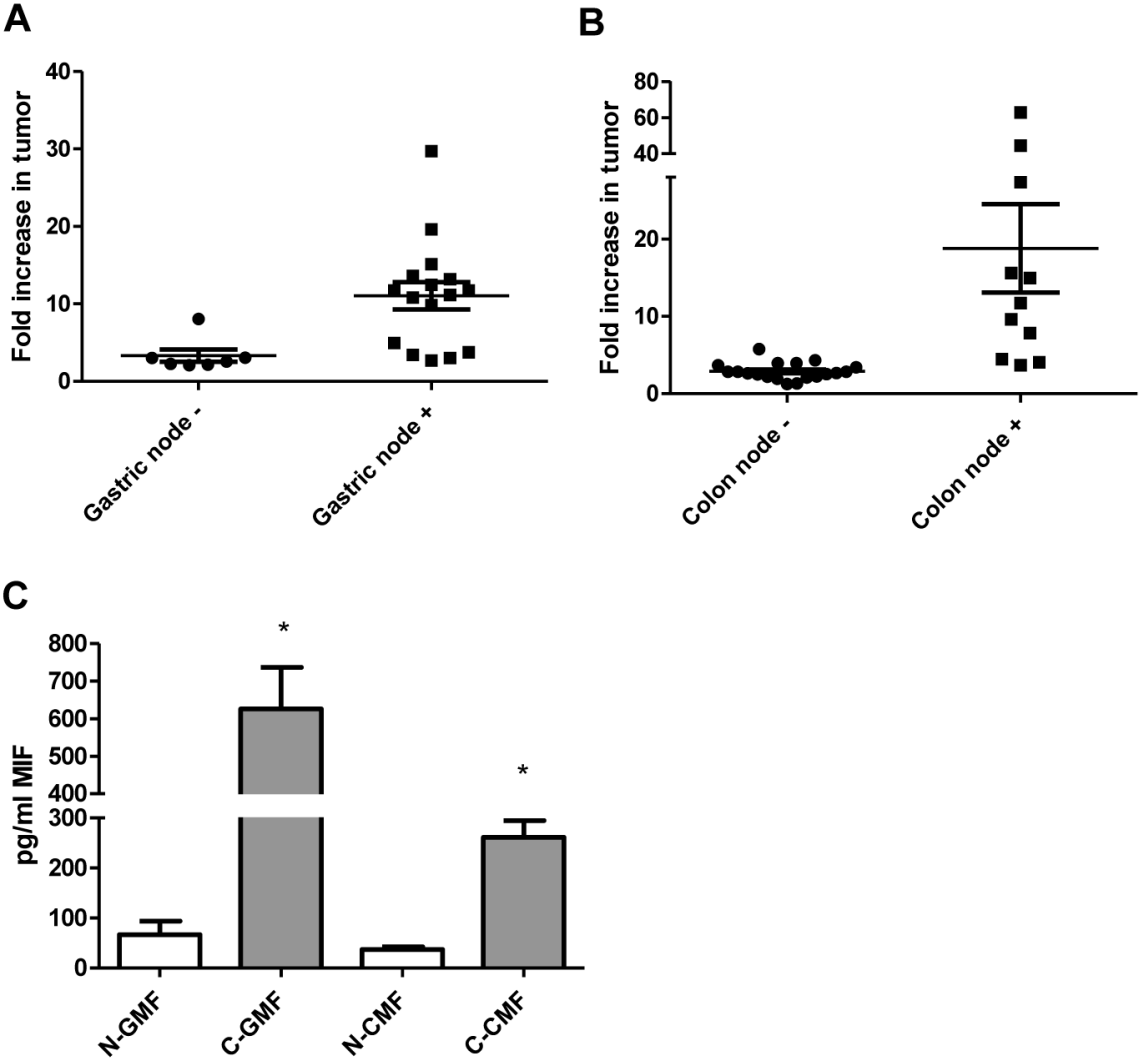
MIF is highly expressed in gastric and colon tumors and human tissue-derived fibroblasts. MIF mRNA levels measured by qRT PCR are increased in A) human gastric tumor samples and at higher levels in tissues from patients with nodal involvement, B) human colon tumor samples and at higher levels in tissues from patients with nodal involvement, and C) in the supernatants tumor derived gastric and colon fibroblasts compared to matched normal as measured by Luminex singleplex assay. N = 8 for C and the mean ± standard error are shown as the results of duplicated in multiple experiments. *p<0.05.

High serum levels of MIF have been suggested to be a diagnostic and prognostic factor for both gastric and colon cancers [6], [17]. To further implicate MIF in gastric and colon cancers, we examined MIF expression in human tumor tissues. The mRNA levels were first examined in a panel of 23 gastric cancer samples and 30 colon cancer samples and compared to normal tissues from the same individual by qRT PCR. Tumor and normal tissue were determined by UNM Pathologists with normal tissues collected as far from tumor margins as possible, a minimum of 5 cm away from the margin. All of the gastric cancer samples exhibited more than 2-fold increase in MIF gene expression while 27 of 30 colon cancer samples exhibited greater than 2-fold increase in MIF gene expression (**Figure 1A and B**). Furthermore, MIF gene expression was compared in samples from patients who did not have nodal involvement to those who did, indicating more aggressive disease. In both gastric and colon cancer, patients who had nodal involvement had substantially higher expression of MIF at the mRNA level. Since epithelial cells have previously been shown to be a source of MIF in gastrointestinal diseases [4], [18], [19], here we also examined cultured fibroblasts cells from human tissues for MIF expression. In previous studies, we and others have developed protocols to process human tissues to establish cultures of gastric and colon fibroblasts from normal and tumor tissues based on CD90 expression [15], [16], [20], [21]. Supernatants from these established cultures were further examined for MIF by Luminex bead assay. As seen in **Figure 1C**, gastric tumor-derived fibroblasts produced approximately 600 pg/ml of MIF while normal tissue-derived fibroblasts produced approximately 70 pg/ml of MIF. Colon tumor-derived fibroblasts produced approximately 260 pg/ml MIF while normal tissue-derived fibroblasts produced approximately 40 pg/ml of MIF. These data suggest that not only is MIF increased in human gastric and colon tumors, but that tumor associated fibroblasts are also a major source of MIF in addition to epithelial cells.

### Human Gastric and Colon Tumor Cell Express Increased CD74

**Figure 2:**
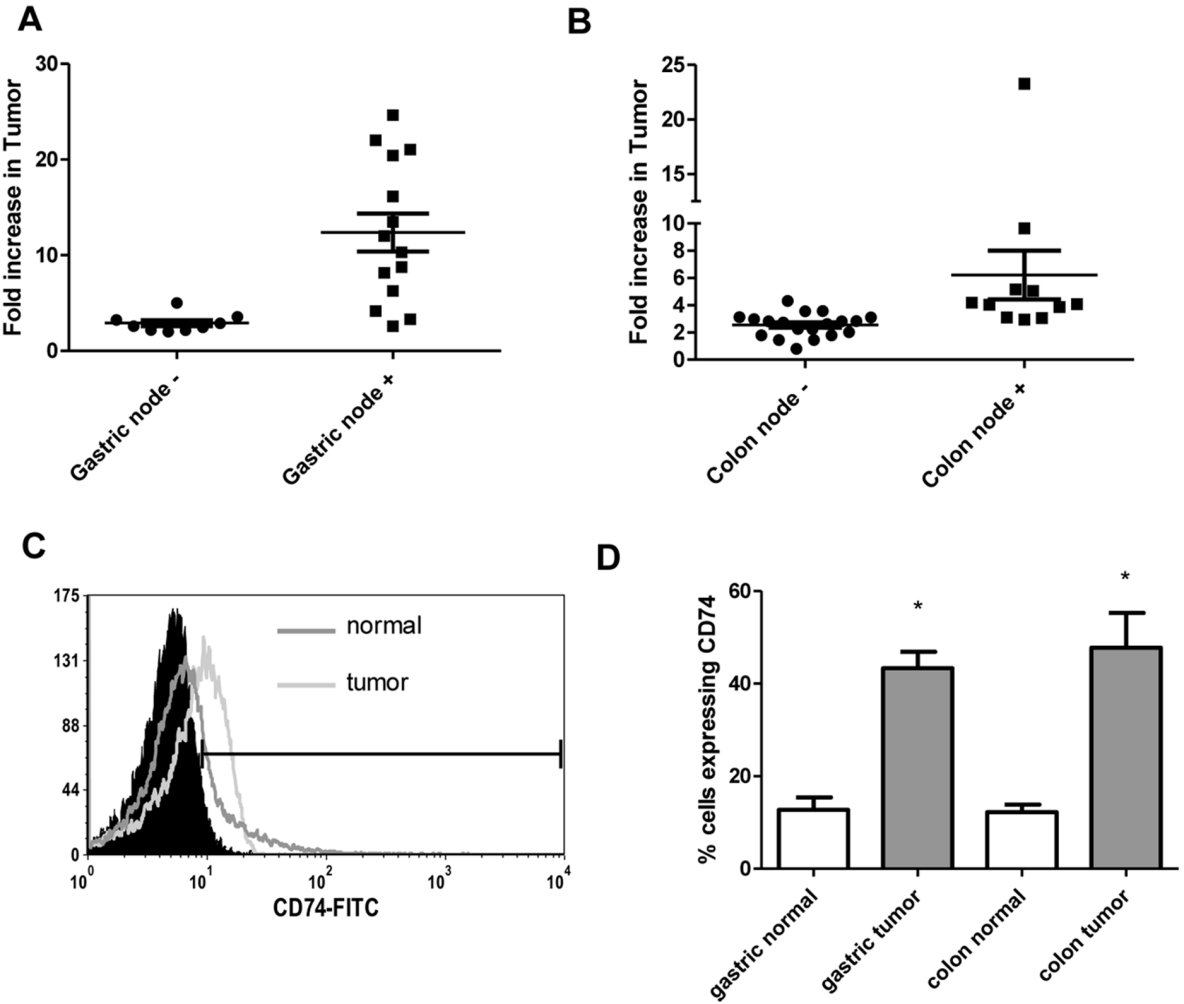
CD74 is highly expressed in human gastric and colon tumors and on isolated epithelial cells. CD74 mRNA levels measured by qRT PCR are increased in A) human gastric tumor samples and at higher levels in tissues from patients with nodal involvement, B) human colon tumor samples and at higher levels in tissues from patients with nodal involvement, and on epithelial cells isolated from human gastric and colon tumors in C) a representative flow cytometry plot and D) in compiled flow cytometry data from all samples. N = 8 for D and the mean ± standard error are shown as the results of duplicated in multiple experiments. *p<0.05.

Since MIF activity requires binding to CD74 [22], we also examined human tumor expression of CD74. Gene expression was examined in the gastric and colon tumor panels and found to be increased by more than 2-fold in all 23 gastric cancer samples and 25 of 30 colon cancer tissues (**Figure 2A and B**). Similar to MIF, the expression level of CD74 mRNA was also substantially higher in samples from patients with nodal involvement indicating worse disease outcome. To further examine epithelial specific expression of CD74, epithelial cells isolated from tumors were stained for cell surface EpCam and CD74 and examined by flow cytometry. After gating on EpCam, CD74 expression was examined. In **Figure 2C**, a representative figure, epithelial cells isolated from a colon tumor have increased CD74 expression compared to epithelial cells isolated from normal tissue. **Figure 2D** shows in compiled data that both gastric and colon tumor epithelial cells show 3–4 times increased expression of CD74 compared to epithelial cells from matched normal tissues. These data indicate that in addition to expressing highly increased levels of MIF human gastric and colon tumor cells express CD74, which is required for MIF activity.

### MIF Induces Proliferation of Gastrointestinal Cells

**Figure 3:**
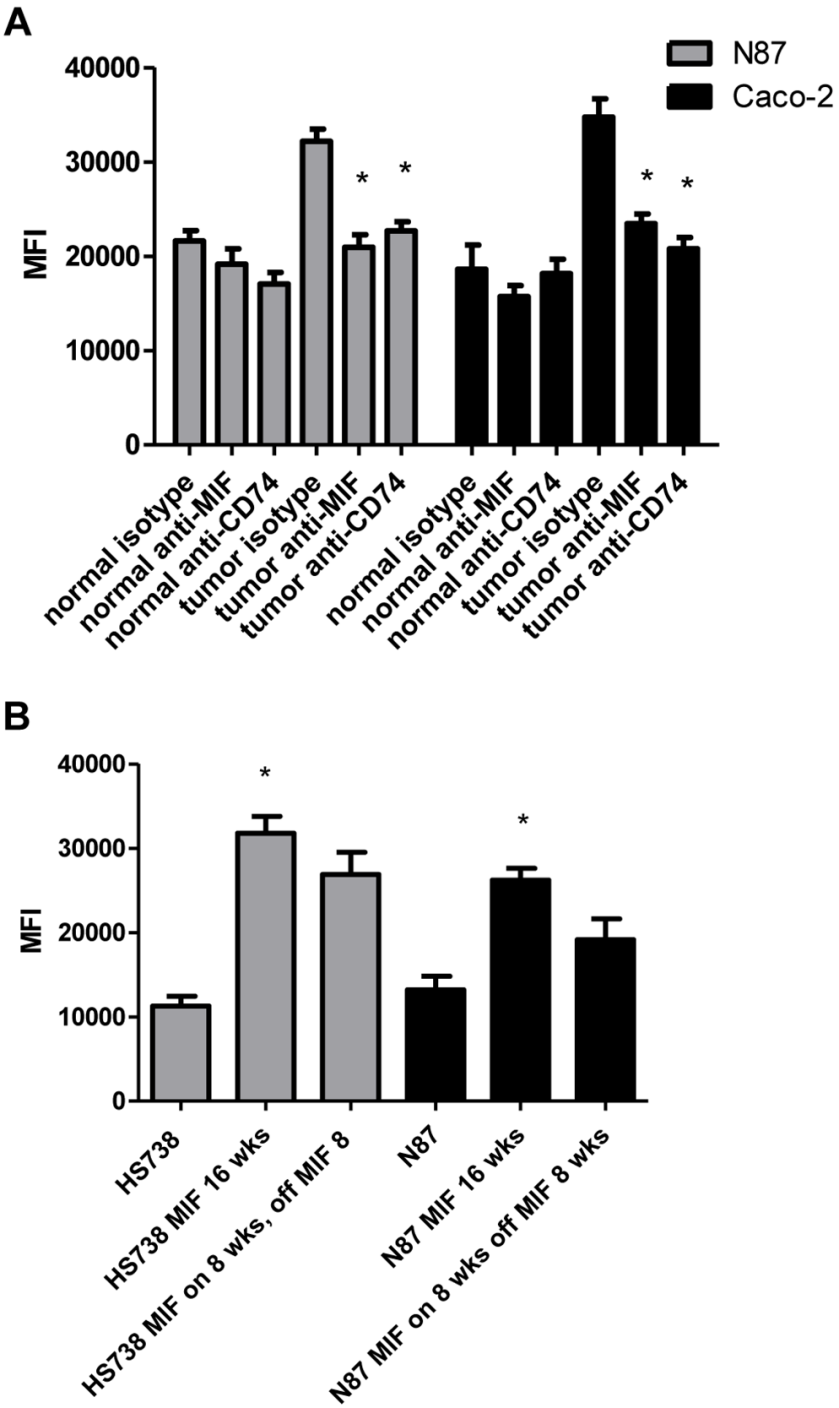
MIF induces proliferation of gastric and colon carcinoma cells. Tumor-derived gastric and colon supernatants induced proliferation of N87 and Caco-2 cells, which was decreased upon adding A) anti-MIF neutralizing antibodies or anti-CD74 blocking antibodies. B) Chronic exposure of HS738 or N87 cells with recombinant MIF increased proliferation that was sustained after returning cells to regular media for 8 weeks. N = 8 and the mean ± standard error are shown as the results of duplicated in multiple experiments. *p<0.05.

MIF is thought to have tumor promoting properties so proliferation was examined here in several approaches. First, N87 gastric carcinoma cells and Caco-2 colon carcinoma cells were incubated with supernatants from cultured normal tissue and tumor-derived fibroblast cells. Proliferation was measured by Cyquant fluorescent proliferation assay for DNA content (**Figure 3A**). Cells incubated with supernatants from tumor-derived fibroblasts showed almost double the proliferation rate as those incubated with supernatants from matched normal tissue-derived fibroblasts. Further, when anti-MIF neutralizing antibodies were added to cultures, proliferation was decreased. A similar result was seen when anti-CD74 blocking antibodies were used. Since our previous work showed that recombinant MIF increases gastric carcinoma cell proliferation [3], to further examine the impact of MIF on cell proliferation in a more chronic setting such as would be the case in gastric and colon cancers, recombinant MIF was added to the media of non-transformed HS738 cells and N87 cells. HS738 are fetal gastric/intestinal cells with fibroblast morphology. Ten ng/ml of recombinant MIF was added to the media of these cells twice a week for up to 16 weeks to model chronic inflammation. A second set of cells was exposed to MIF for 8 weeks and then regular media for 8 weeks. In **Figure 3B**, cells exposed to MIF show 2 to 3 fold increased proliferation rates. Increased proliferation was maintained by cells that had MIF treatment for 8 weeks and then MIF removed for 8 weeks suggesting a lasting effect on cells chronically treated with MIF.

### Chronic MIF Treatment Induces and Maintains Pro-tumorigenic Signaling

**Figure 4:**
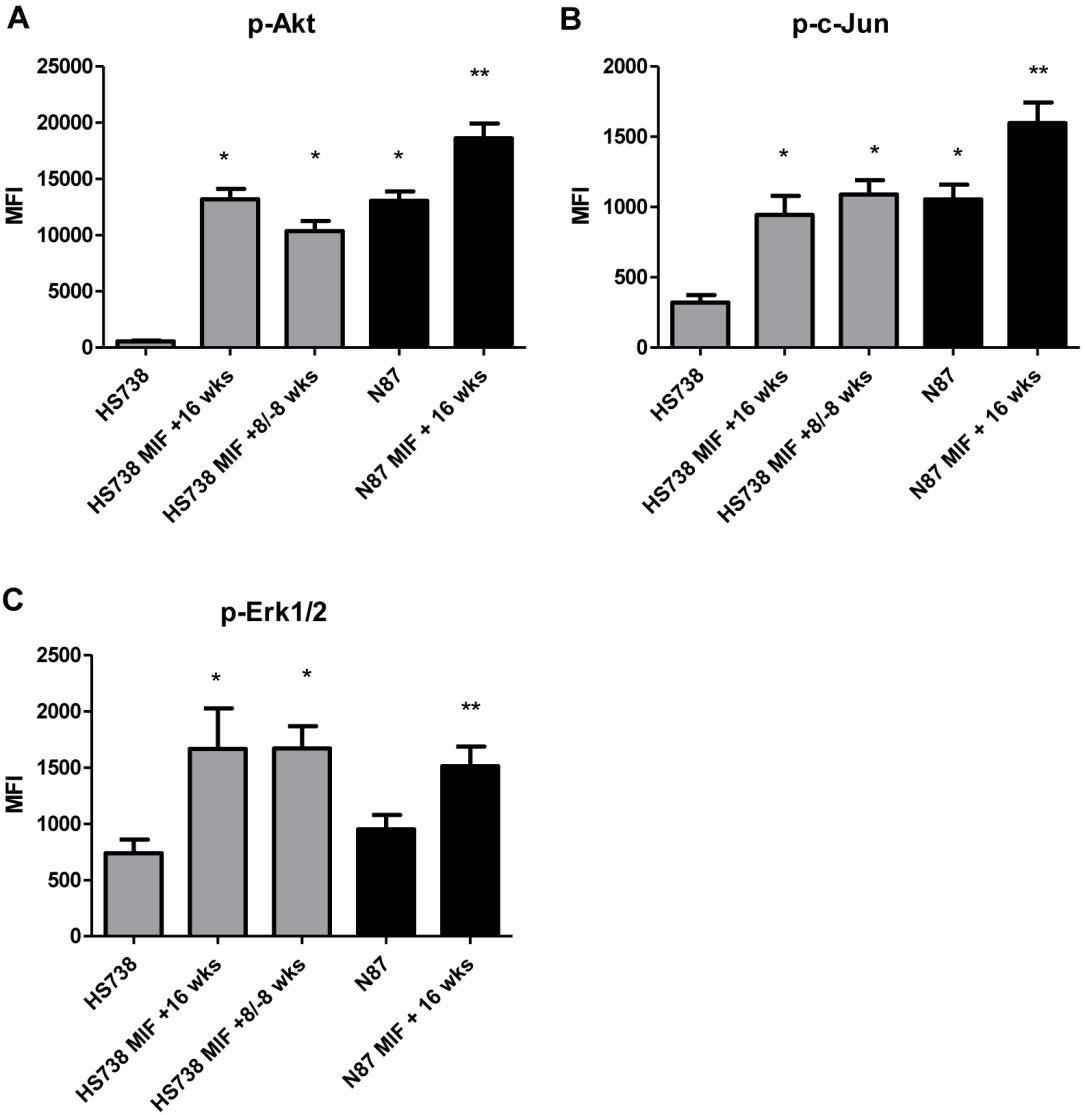
MIF induces pro-tumorigenic signaling. Chronic MIF treatment induces A) Akt, B) c-Jun, and C) Erk1/2 phosphorylation. N = 8 and the mean ± standard error are shown as the results of duplicated in multiple experiments. *p<0.05.

Given the impact of MIF on sustained proliferation of HS738 and N87 cells, the mechanism by which proliferation is sustained in these cells was examined. Lysates of untreated cells and cells treated chronically with MIF were normalized for total protein levels and run on phosphoprotein bead arrays. Phospho-Akt, c-Jun, and Erk1/2 were examined as pathways MIF may induce. Phosphorylated Akt was highly induced in MIF treated cells treated for 16 weeks with MIF, going from a mean fluorescence intensity (MFI) of 550 in untreated cells to 13200 in MIF treated cells (**Figure 4A**). HS738 cells exposed to MIF for 8 weeks with subsequent 8 weeks of regular media sustained increased Akt phosphorylation at an MFI of 10363. MIF treatment of N87 cells also showed increased Akt phosphorylation as control cancer cells, which was decreased upon removal of MIF. Similar patterns were seen with phosphorylated c-Jun (**Figure 4B**) where untreated cells had an MFI of 320 while treated cells showed and increase to an MFI of approximately 950, which was sustained when cells were returned to media without MIF. Increased phospho-c-Jun was also seen upon N87 chronic treatment with MIF as a control, but showed some decrease upon return to media without MIF. Erk1/2 phosphorylation again showed a similar pattern (**Figure 4C**) with treated cells showing an increase in MFI from 730 to 1660. High basal levels of phosphorylated Akt and c-Jun were seen in N87 suggesting these pathways are already activated in N87. MIF also increased activation of these pathways in N87. These results suggest that chronic MIF treatment induces sustained pro-tumorigenic signaling in GI cells.

### Chronic MIF Treatment Induces Mesenchymal Epithelial Transition of GI Fibroblast Cells

**Figure 5:**
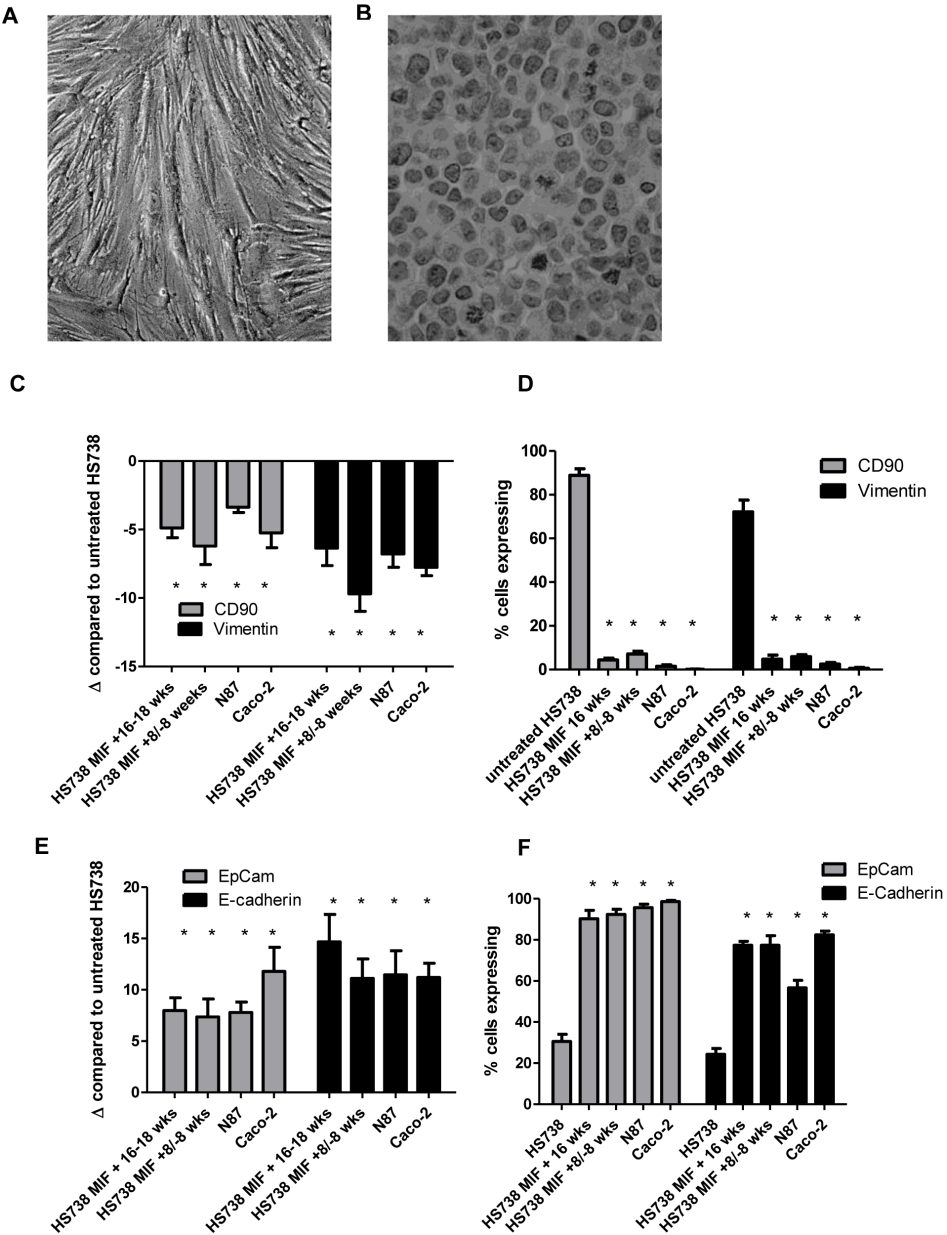
Chronic MIF exposure induces mesenchymal epithelial transition. HS738 cells have A) a fibroblast morphology, which changes B) upon chronic exposure to recombinant MIF. MIF exposure also decreases expression of fibroblast markers by C) mRNA levels by qRT PCR compared to untreated cells and by D) flow cytometry, while increasing expression of epithelial markers EpCam and E-cadherin by E) mRNA levels by qRT PCR compared to untreated cells and by D) flow cytometry. N = 8 and the mean ± standard error are shown as the results of duplicated in multiple experiments. *p<0.05.

In addition to increased proliferation and pro-tumorigenic signaling, MIF induced changes in morphology in HS738 cells. As seen in **Figure 5A and B**, chronic MIF treatment resulted in a change from a fibroblast morphology to more of an epithelial morphology. To further investigate this, we examined expression of fibroblast and epithelial markers. By qRT PCR, we found HS738 chronically treated with MIF to decrease expression of fibroblast markers vimentin and CD90. CD90 was decreased by up to 6-fold in cells chronically treated with MIF for 16–18 weeks compared to HS738 grown in media without MIF (**Figure 5C**). These cells were compared to N87 and Caco-2 as epithelial controls, which also showed 3 to 5-fold decreases in CD90 mRNA levels compared to HS738 grown in media without MIF. Vimentin was also decreased by up to 9.6 fold in MIF treated cells and up to 7-fold in N87 cells. These changes were maintained in cells that were exposed to MIF for 8 weeks and then had MIF removed for 8 weeks. CD90 and vimentin expression were also examined by flow cytometry and found to be dramatically decreased (**Figure 5D**). Over 80% of HS738 cells showed expression of CD90, but expression was decreased to 5% after chronic MIF treatment. Similar results were seen with vimentin staining with over 70% of HS738 staining positive, which was decreased to less than 5% with chronic MIF treatment. Since these results indicate a loss of fibroblast markers, epithelial markers were also examined. EpCam and E-cadherin gene expression levels were examined by qRT PCR and MIF treated cells were found to be increased in EpCam mRNA by approximately 8-fold compared to untreated HS738, which was similar to N87 and Caco-2 epithelial controls (**Figure 5E**). E-cadherin gene expression was examined as a second marker and found to be upregulated by 14.7-fold in MIF treated HS738 and 11-fold in the N87 and Caco-2 epithelial controls. These epithelial markers were also examined by flow cytometry (**Figure 5F**). Approximately 30% of HS738 cells were found to express EpCam, which was increased to 90% with chronic MIF treatment and 96% by epithelial control cells. E-cadherin was expressed by 24% of HS738, and was increased to 77% with MIF treated cells and 57% with N87 cells. Taken together, these results suggest that chronic MIF treatment induces morphology changes, downregulation of fibroblast markers, and upregulation of epithelial markers suggesting mesenchymal epithelial transition of HS738 cells.

### Chronic MIF Treatment Induces Cell Transformation of GI Fibroblast Cells

**Figure 6:**
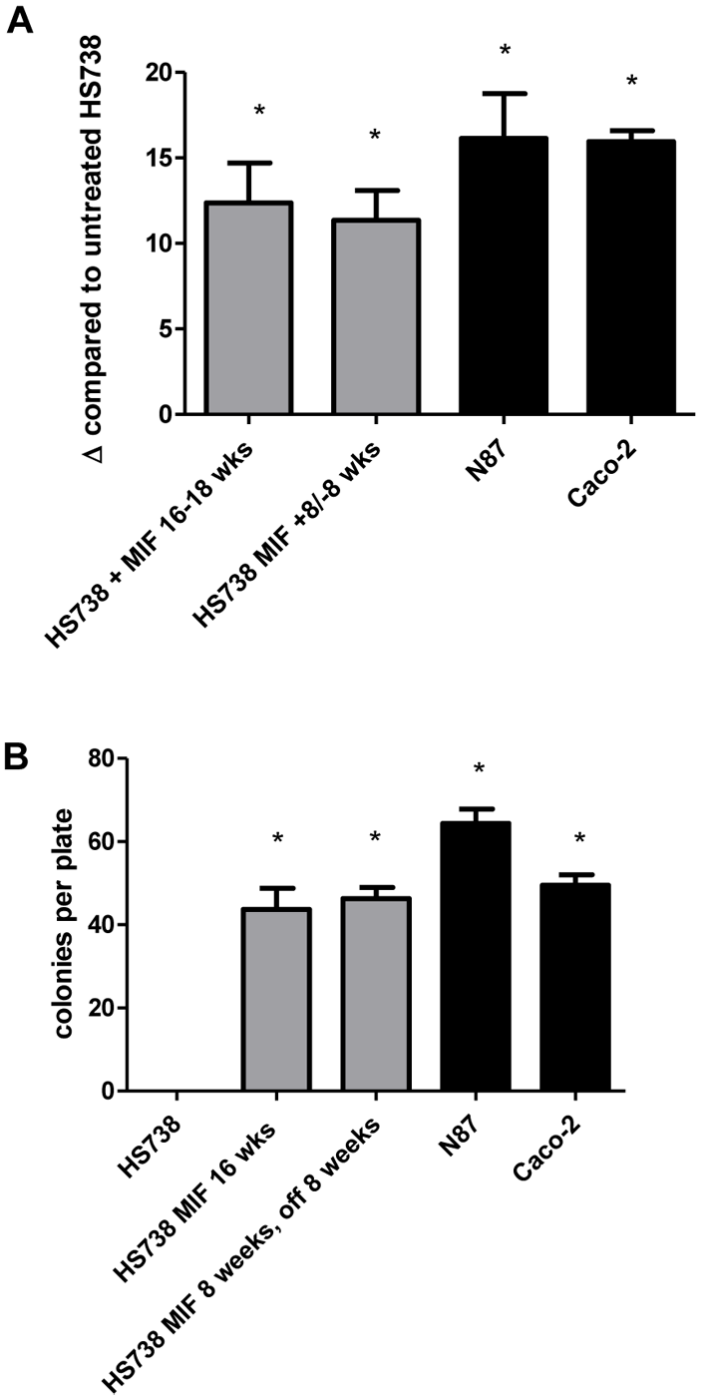
Chronic MIF exposure induces HS738 cell transformation. HS738 chronic exposure to MIF induces A) upregulation of TERT gene expression so similar levels as N87 control cells and B) colony formation in a focus forming assay. N = 8 and the mean ± standard error are shown as the results of duplicated in multiple experiments. *p<0.05.

Given the increased proliferation and transition from cells expressing fibroblast markers to cells expressing epithelial markers, cell transformation was examined in HS738 chronically treated with MIF. This was first examined by measuring telomerase reverse transcriptase (TERT) gene expression since upregulation of TERT is known to be associated with cell immortalization [23], [24]. In **Figure 6A**, HS738 cells exposed to MIF for 16 weeks show a 12-fold increase in TERT gene expression compared to HS738 in regular media. HS738 cells treated with MIF for 8 weeks and then regular media for 8 weeks retained the increase in TERT gene expression. N87 were examined as a positive control and showed a 16-fold increase in TERT expression compared to HS738 in regular media. For another measure of cell transformation, focus forming assays were performed. Untreated and MIF treated cells were plated at 10^3^ for 2 weeks, stained with hematoxylin, and colonies were counted. HS738 were found not to form colonies, while HS738 treated with MIF for 16 weeks formed an average of 40 colonies per plate (**Figure 6B**). Similar results were seen with HS738 treated with MIF for 8 weeks and then regular media for 8 weeks with N87 and Caco-2 control cells. These results suggest that HS738 treated chronically with MIF may be transformed and resemble carcinoma cells such as N87.

## Discussion

MIF was first characterized in the 1960s for a role in enhancing macrophage function [25]. While initially thought to only come from T cells, MIF has more recently been shown to have multiple sources including macrophages themselves [26]. When first described, MIF was shown to inhibit macrophage mobilization to maintain these cells at a site of injury or infection. MIF later emerged as an innate mediator of chronic inflammatory diseases such as atherosclerosis and arthritis [27], [28]. As the examination of MIF in chronic inflammation grew, it became a focus in infectious disease as well [29]–[32]. Our work previous work also showed a role for MIF and CD74 in inflammation and pro-tumorigenic signaling during *H. pylori* infection [3], [4]. Other groups have also started to suggest that MIF may have diagnostic and prognostic value in inflammation-associated cancers [6], [8], [33], [34].

In 2003, MIF was found to bind to CD74 as a primary receptor in order to initiate signaling events [22]. This initial, impactful finding showed that MIF induced cell proliferation through the MAP kinase cascade. Continuing studies suggested that CD74 couples with CXCR2 or CXCR4 to induce signaling events [18], [35]. Further studies also found a role for the Akt pathway in general cell survival studies [36], [37]. Additionally, one recent study showed that MIF regulates Akt signaling in a hypoxic environment to activate mesenchymal stem cells to support proliferation [38]. Our previous studies followed up with a role for EGFR transactivation in tumor cells that enhanced proliferation of gastric cancer cells [4]. In that study, we also showed that the effects of MIF on transactivation of EGFR in HS738 and N87, the same cells used in this study, could be decreased by blocking the initial binding partner of MIF, CD74, or also by blocking the co-receptors for CD74, CXCR2 and CXCR4. Others have also shown that CXCR2 and CXCR4 are expressed on the colon cancer cell lines used here [39], [40]. Here we also show that chronic exposure of normal fibroblast cells to MIF induces Erk1/2, Akt, and C-jun signaling, which is maintained after removing MIF from the media. Thus, is the first study to show chronic effects of MIF on proliferative signaling. In this regard it is not surprising that MIF may be an important link between chronic inflammation and tumorigenesis. Here we found that exposure of HS738 cells to MIF for 8 weeks led to a sustained increase in proliferation that was maintained even after returning the cells to media without MIF. The impact of MIF in cancer cell proliferation was also shown by another group that inhibited MIF production in pancreatic cancer cells using siRNAs and found not only a decrease in proliferation, but also the induction of apoptosis of these cells [41].

Recent evidence suggests that the interplay between epithelial cells and various cells of the tumor microenvironment play a major role in tumor development and progression. Our work has focused on fibroblasts/myofibroblasts as major players in inflammatory diseases of the GI tract. Since we previously showed that HS738 produce MIF in response to *H. pylori* as a potential link between inflammation and gastric cancer, here we further examined tumor associated fibroblasts for production of MIF. We found MIF to be highly produced by these cells compared to those from uninvolved tissues from the same patient in both gastric and colon cancers. Pro-inflammatory mediators such as MIF may be upregulated in stromal cells in an autocrine manner or a paracrine manner during crosstalk between stromal cells and tumor cells. Additionally, MIF was shown to be upregulated in synovial fibroblasts in response to the IFN-γ, CD40 ligand, IL-15, IL-1β, TNF-α, and TGF-β [42]. Several of this mediators are also thought to be upregulated during increased during *H. pylori* infection and gastric cancer [16], [43]. Thus, stromal cell production of MIF may have an important impact in the tumor microenvironment.

Furthermore, we found that chronic exposure of HS738 cells that express CD90 and vimentin fibroblast markers, to downregulate these markers in conjunction with upregulating the epithelial markers, EpCam and E-cadherin. These data along with the drastic change in morphology seen by MIF treated cells suggest that chronic MIF treatment induces mesenchymal epithelial transition of fibroblasts cells. In the tumor microenvironment, this could have an important impact on not only tumor growth, but also in a metastatic setting. One group has shown that MIF also promotes fibroblast migration in a scratch would assay suggesting further metastatic potential of this cytokine in conjunction with enhancing MET [44]. In addition to MET, we also found the cells chronically treated with MIF to undergo processed leading toward cell transformation. These included the upregulation of TERT and colony formation in a focus forming assay. These data further support a critical role for MIF in inflammation-associated cancers.

We also found that patient gastric and colon tumors expressing the highest levels of MIF and CD74 to be from patients with nodal involvement, thus suggesting an association with more aggressive disease. These data are supported by another study that shows higher MIF expression by tumor cells from nasopharyngeal carcinoma patients correlated with advanced clinical stage of disease [45]. A few studies have suggested that MIF may be involved with tumor progression, one showing that MIF induces invasion of colon cancer cells in vitro in assays using matrigel [46]. This study also showed in mice that inhibition of MIF decreased metastasis of tumor cells injected into the tail vein. The potential for MIF to be involved in proliferation, invasion, MET, and transformation in our study and others suggest that MIF blockade should be examined as a potential tumor target in inflammation-associated cancers. Several studies have tested MIF inhibitors or MIF blocking antibodies to decrease inflammation in diseases such as sepsis and autoimmune diabetes [25], [47], and thus, the same approach could be tested in colon cancer.
